# Gloss retention of direct composites and corresponding CAD/CAM composite blocks

**DOI:** 10.1002/cre2.505

**Published:** 2021-10-14

**Authors:** Stefano Ardu, Olivier Duc, Ivo Krejci, Emilie Bétrisey, Enrico Di Bella, René Daher

**Affiliations:** ^1^ Treatment Plan Unit and Division of Cariology & Endodontology, University Clinics of Dental Medicine University of Geneva Geneva Switzerland; ^2^ Division of Cariology & Endodontology, University Clinics of Dental Medicine University of Geneva Geneva Switzerland; ^3^ Department of Economics and Quantitative Methods University of Genoa Genoa Italy

**Keywords:** CAD/CAM, gloss resin composite

## Abstract

**Objectives:**

To compare gloss retention of four different resin composites with their corresponding CAD/CAM composite blocks.

**Materials and methods:**

Four direct resin composites (Filtek Supreme XTE A2 Body (3M, USA), Tetric EvoCeram A2 (Ivoclar Vivadent, Liechtenstein), GrandioSO x‐tra A2 (VOCO, Germany), G‐aenial Universal A2 (GC, Japan)), and their corresponding CAD/CAM composite blocks were tested. A total of 288 samples were prepared and three different tests were performed: brushing, exposition to acidic fluoride gel and exposition to alcoholic solution. Gloss values were obtained by means of a glossmeter at T0 before aging and T60 after 1 h of aging.

**Results:**

Mean gloss values ranged from 0.9 after brushing tests to 79.0 after the alcohol test witnessing a high gloss variability depending on the materials and the aging test. Statistical analysis by means of two‐way repeated measures ANOVA followed by Fisher's LSD post‐hoc test revealed significant differences between materials, storage media, and their interactions.

**Conclusion:**

Gloss retention seems to be dependent on the composite type (direct or CAD/CAM block) and composite brand and varies in respect to the type of aging. CAD/CAM materials showed a higher resistance toward alcohol exposure.

## 1 INTRODUCTION

Besides being the material of choice for direct restorations (Ardu & Krejci, 2006; Dietschi et al., 2012) composite resins in the form of prefabricated blocks are rapidly invading indirect CAD/CAM workflows. Most manufacturers have therefore launched the equivalent of their direct resin composites in the form of CAD/CAM blocks due to the multiple advantages of this type of materials, such as ease of repair, perfect compatibility with adhesive techniques, higher resilience compared to brittle ceramics, relatively lower cost, and sufficient mechanical properties for single‐tooth restorations (Ardu et al., 2011; Dietschi et al., 2019; Jassé et al., 2013). Furthermore, the use of CAD/CAM composites for inlays, onlays, endocrowns, and even for anterior restorations allows for a decrease of general costs by cutting down dental laboratory expenses in the medium to the long term view (da Veiga et al., 2016; Dietschi et al., 2019), despite the initial investment of the acquisition and milling machines.

Composite CAD/CAM blocks are often made out of the same or very similar components as their respective light‐cured direct restorative resin composites, with the difference that the curing step is performed by the manufacturers rather than clinically by the dentist. This well‐controlled professional curing under high pressure and high temperature leads to a higher conversion rate and to even, as claimed by some manufacturers, better clinically performing materials (Batalha‐Silva et al., 2013). Enhanced properties are not limited to the mechanical aspect, but also to the optical and chemical stability, which reduces staining and increases gloss retention. More specifically, gloss retention is an important factor as it allows for a better esthetic appearance of composite restorations, especially in anterior area. A high lip line can cause, in fact, a reduced amount of saliva on the tooth surface, causing a progressive dull aspect of the restored tooth, especially if it is located between intact natural teeth (Lefever et al., 2012). Exposure of resin composites in general, to acids, alcohol as well as brushing habits is known to alter their appearance on the long term, but no data exists on the comparison between the gloss retention of recently launched CAD/CAM composites and their corresponding traditional direct resin composites.

Therefore, the aim of this in vitro study was to compare the gloss retention of four restorative direct resin composites, and their respective indirect CAD/CAM blocks after challenging their surfaces with chemical and mechanical attacks. The null hypothesis was that mechanical and chemical agents were not able to significantly decrease surface gloss of direct composite resin materials as well as indirect CAD/CAM blocks.

## 2 MATERIALS AND METHODS

A total of 288 samples were prepared, specifically 12 samples for each tested material and for each aging test (Table 1). The choice of materials was based on commonly used light‐cured resin composites together with their respective CAD/CAM blocks. The manufacturers were also asked to confirm that the light‐cured composites and their respective CAD/CAM blocks had a similar composition. A nano‐filled composite, Filtek Supreme XTE, a hybrid composite with pre‐polymerized particles, Tetric EvoCeram, a highly filled hybrid composite, GrandioSO x‐tra and a newly developed hybrid composite with pre‐polymerized particles and diffused fumed silica fillers, G‐aenial Universal were chosen as direct resin composite references. For each of the four direct light‐curing resin composites, group FS, Filtek Supreme XTE A2 Body (3M, USA), group TE, Tetric EvoCeram A2 (Ivoclar Vivadent, Liechtenstein), group GS, GrandioSO x‐tra A2 (VOCO, Germany), group GU, G‐aenial Universal A2 (GC, Japan), samples of 8 mm diameter were prepared by filling a 1.1 mm high cylindrical mold and gently pressing the resin composite with a transparent polyester strip (Hawe Transparent Strips, Kerr, Orange, CA, USA) and a glass slide. The resin composites were then light cured for 20 s by using a high power LED light curing unit (LCU) (L.E.Demetron II, Kerr), placed in contact with the 1 mm glass slide, at an irradiance of 1200 mW/cm^2^ that was measured with a LED radiometer (Demetron, Kerr). Subsequently, all samples were manually polished up, from the measuring side, for 30 s each to the finest polishing disc (Coarse, Medium, Fine, Superfine Sof‐Lex™, 3M, USA) in order to achieve the final thickness of 1 mm. All discs were discarded after each specimen polishing. Operator‐dependent application factors in direct composites were reduced by applying the material in standardized molds and by pressing the surface with a flat glass slide to obtain similar samples. For the CAD/CAM blocks, group LU, Lava Ultimate A2 LT (3M, USA, USA), group TC, Tetric CAD A2 (Ivoclar Vivadent, Liechtenstein), group GCAD, Grandio CAD A2 (VOCO, Germany), group CS, Cerasmart 270 A2 LT (GC, Japan), slices of 1.1 mm were cut by means of a low‐speed microtome (Miniton Fuse 2.5AT, Struers, Copenhagen, Denmark) under profuse water. Each slice was then manually reduced by the same polishing discs system mentioned above to reach the 1 mm thickness, by the same operator and under the same conditions of the direct resin composite groups to reduce variations during this step. All samples were stored at 37°C for 7 days in an incubator (INP‐500, Memmert GmbH, Büchenbach, Germany) then gloss was measured at T0 by means of a glossmeter (Novo‐Curve, Serial No. NOFF06090068, Rhopoint Instrumentation, Bexhill on Sea, UK).

**Table 1:** Tested materials with their composition, respective lots, and expiry dates

Composite resin lot and exp date	Composition	CAD/CAM resin	Composition	Manufacturer
Filtek Supreme XTE A2 Lot: N9874223 Exp: 2021‐04‐28	Bis‐GMA, UDMA, TEGDMA, bis‐EMA silica filler, zirconia filler, aggregated zirconia/silica clusters	Lava Ultimate A2 LT Lot: N934623 Exp: 2022‐07‐28	SiO_2_ fillers, ZrO_2_ ZrO_2_/SiO_2_ nanoclusters, bis‐GMA, UDMA, bis‐EMA, TEGDMA matrix	3M, USA
Tetric EvoCeram A2 Lot: X4140 Exp: 2022‐07‐20	Urethane dimethacrylate bis‐GMA ytterbium trifluoride ethyoxylated bisphenol A dimethacrylate, barium glass filler, ytterbium trifluoride, mixed oxide	Tetric CAD A2 MT Lot: X46766 Exp: 2021‐12‐30	Dimethacrylates, bis‐GMA, bis‐EMA, TEGDMA, UDMA, barium glass silicon dioxide	Ivoclar Vivadent, Liechtenstein
GrandioSO x‐tra A2 Lot: 1805358 Exp: 2020‐04	Bis‐GMA, bis‐EMA, Teg DMA, inorganic filler, organically modified silica	Grandio CAD A2 LT Lot: 1751258 Exp: 2022‐08	UDMA, Teg DMA, nanohybrid fillers	VOCO, Germany
G‐aenial Universal A2 Lot: 181012A Exp: 2021‐10‐11	UDMA, bis‐MEPP, TEGDMA silicon dioxide, strontium glass	Cerasmart 270 A2 LT Lot: 1805101 A3 14L LT	UDMA, bis‐MEPP, DMA, barium glass, SiO_2_ nanoparticle	GC, Japan

This device uses as measurement method a specular gloss at a 60° incident angle over a 2 mm × 2 mm area by using a source‐filter photocell combination that is spectrally corrected to obtain CIE luminous efficiency with CIE source C with an exposure time of 2 s.

Within each of the eight restorative materials, the 36 samples were randomly divided into three equal groups for the three aging tests. Specifically, in order to measure gloss of the samples, according to Heintze et al. (2006) three gloss measurements per sample were done (1 every 120° of samples rotation). A complete recalibration of the glossmeter with the calibration plate provided by the manufacturer was done after each tested group, in order to avoid bias and in accordance with other studies (Ardu et al., 2009; Da Costa et al., 2007; Lefever et al., 2014).

Subsequently all samples were aged either for 1 h in a 75% ethanol aqueous solution (Merck, Darmastadt, Germany) for the alcohol test, or for 1 h in amino fluoride gel (Elmex gelée, Gaba) for the Elmex gelée test or for 1 h of brushing by means of a toothbrush (Curaprox 1560 Soft, Kriens, Switzerland) and toothpaste (Signal Anti‐Caries, Unilever Schweiz GmbH, Thayngen, Switzerland) where 1.5 g of toothpaste was mixed with 5 ml of water in a brushing simulator (Zahnburstsimulator ZM 3.12, SD Mechatronik GmbH, Rosenheim, Germany) for the brushing test and then measured by a glossmeter for a second time (T60).

The comparison between initial and final gloss values for the different materials and storage media were statistically evaluated by means of a two‐way repeated measures ANOVA followed by Fisher's LSD post‐hoc tests. Normality assumptions have been tested by means of Kolmogorov–Smirnov test (normality assumption criteria).

## 3 RESULTS

After manual polishing, mean gloss values ranged from 56.2 (GS) to 73.4 (GCAD). After brushing test, mean gloss values ranged from to 0.9 (GS) to 50.0 (TC). After the Elmex gelée test, mean gloss values ranged from 19.7 (GCAD) to 45.0 (CS). After the alcohol test, mean gloss values ranged from 52.8 (GS) to 79.0 (LU).

Gloss retention evaluated by means of repeated measures two‐way ANOVA followed by Fisher's LSD post‐hoc tests revealed statistically significant differences among all the materials, aging tests (except for the alcohol group) and their interactions (*p*‐values <0.01). Results also showed significant differences between the group means (*p*‐values <0.01): (1) for the brushing test, FS, TC, and GU had higher gloss values than the other tested materials; (2) for the Elmex gelée test, FS, TC, TE, and CS had higher gloss values than the other tested materials; (3) for the alcohol test, LU had higher gloss values than the other tested materials. Materials' initial and final values for the three aging tests and the control group are illustrated in Table 2 along with the rankings after aging.

**Table 2:** Materials' initial and final values for the three aging tests and materials' rankings after aging tests, where A is the best and D is the worst

	Aging test							
	Brushing		Elmex gelée		Alcohol		Distilled water	
	Initial mean and SD	Final mean and SD	Initial mean and SD	Final mean and SD	Initial mean and SD	Final mean and SD	Initial mean and SD	Final mean and SD
Filtek Supreme	69.88 (6.35)	47.88 (5.28)^A^	68.64 (6.91)	37.70 (7.05)^A^	65.52 (8.08)	71.74 (2.9)^B^	71.86 (5.45)	72.03 (3.09)^A^
Lava Ultimate	69.35 (3.11)	38.54 (3.6)^B^	71.17 (4.44)	31.43 (6.65)^B^	72.49 (6.48)	79.00 (4.85)^A^	70.69 (5.07)	71.64 (3.68)^A^
Tetric CAD Refill	72.27 (4.28)	49.95 (3.25)^A^	66.33 (2.88)	38.46 (7.45)^A^	69.32 (5.23)	68.39 (4.96)^B^	68.44 (4.42)	69.02 (3.88)^A^
Tetric EvoCeram	71.67 (3.04)	38.82 (2.8)^B^	68.48 (1.83)	38.74 (5.64)^A^	63.65 (3.74)	58.15 (4.5)^D^	72.13 (1.52)	71.34 (2.52)^A^
Grandio blocs LT	73.86 (4.79)	4.83 (2.9)^C^	72.11 (3.11)	19.74 (5.31)^C^	74.29 (3.73)	67.24 (5.36)^B^	72.99 (3.81)	73.34 (2.99)^A^
GrandioSO x‐tra	54.25 (4.13)	0.85 (0.25)^C^	55.94 (4.4)	21.71 (8.72)^C^	58.46 (4.32)	52.84 (3.4)^D^	51.68 (2.42)	50.48 (2.09)^D^
Cerasmart 270	62.89 (3.2)	36.67 (5.35)^B^	65.29 (3.32)	45.05 (12.4)^A^	61.71 (2.64)	62.59 (2.75)^C^	63.22 (3.01)	63.44 (2.86)^C^
G‐aenial Universal	72.52 (5.39)	49.59 (4.16)^A^	74.41 (4.02)	36.21 (16.03)^B^	72.59 (4.18)	66.56 (3.86)^B^	70.93 (4.77)	69.13 (2.83)^B^

## 4 DISCUSSION

The aim of this in vitro study was to compare the gloss retention of four restorative direct resin composites, and their respective indirect CAD/CAM blocks after challenging their surfaces with chemical and mechanical attacks. These common clinically relevant aging factors (Ardu et al., 2009; Goldstein & Lerner, 1991; Lefever et al., 2012, 2014; Neme et al., 2003; Tanoue et al., 2000), that is, brushing, exposition to acidic fluoride gel, and exposition to alcoholic solutions, may be present in routine diets and common oral hygiene habits and could highly influence the esthetic appearance of direct and indirect CAD/CAM composite restorations.

In our study design, we decided to go for manual polishing technique that was intentionally used in order to represent clinical reality. This approach led to lower gloss values compared to results obtained by machine‐polished samples with up to 4000 grit size paper (Lefever et al., 2012, 2014). The aging tests were standardized by either submerging the samples in the same medium for the alcohol and fluoride gelée tests and by using an automated brushing simulator. To avoid any subjective bias in the evaluation of the surface gloss, the use of a glossmeter that has the capacity to numerically report surface reflectance of a restricted area under standardized conditions was used. This allows to reduce confounding factors such as angle of the observer and illumination (Sheen Instruments Ltd, 2000) which was set at 60° for all measurements, in accordance with Da Costa et al. (2007).

The alcohol test was performed to evaluate the effect of possible softening by alcohol on the gloss of the composite surface. A solution of 75% ethanol was used according to previous studies (Ardu et al., 2009; Yap et al., 2003). Condon and Ferracane (1997) showed that aging through ethanol storage (75% ethanol aqueous solution, 37°C) produces an increase in subsequent wear only in composite materials that are under‐cured, while no effect should be detected in well polymerized samples. The mode of action of alcohol is related to its intrinsic amphiphilic nature, which increases water sorption of the hydrophilic part of the composites, such as the resin fraction for example. The accompanying volume increase can alter micro‐morphology of the surface resulting in lower gloss refraction, as witnessed in the present in vitro experiment. A general decrease of gloss values was detected for all the tested materials with the only exception of the nano‐filled composites (i.e., Lava Ultimate and Filtek Supreme) that showed higher gloss values after having been challenged with alcohol. This could be due to the protective effect from OH‐groups of bis‐EMA which is present in the matrix. CAD/CAM blocks on the other hand, most probably due to their higher degree of conversion, performed better when compared with their respective light‐cured homologues, which confirms the Condon and Ferracane observations. The only exception was seen in the G‐aenial Universal/Cerasmart pair where already before the test, the light‐cured direct material showed better gloss values than the corresponding CAD/CAM block. The data for this pair also show a significant decrease of gloss of the light‐cured material after the test while the gloss values of the CAD/CAM blocks remained relatively stable. It could be hypothesized that if the tests were to be extended, the CAD/CAM material could have reached a higher gloss value than the respective light cured one.

In order to mimic acidic attacks, Elmex gelée was used as proposed by Ardu et al. (2009) due to the presence of highly concentrated aminofluoride. This kind of gels is widely and regularly employed in the field of caries prevention due to its presumed anticaries capacity. This formulation, when in contact with water, develops hydrofluoric acid and becomes quite aggressive against glass and ceramics as well as composite fillers (Wozniak et al., 1991) which leads to structural changes altering the gloss behavior. All tested materials were highly affected by the aminofluoride gel and no superiority of CAD/CAM blocks was found over the light‐cured materials. The results showed that the direct composite FS was slightly better in the FS/LU pair, while the CAD/CAM block CS was slightly better in the GU/CS pair, and that no difference existed in the two remaining pairs.

Concerning the brushing test, the same protocol was used as in previous studies (Hanasaki et al., 2018; Lefever et al., 2012, 2014; Wiegand et al., 2013) where the influence of each test parameter is widely discussed. Specifically, in this study, a medium abrasive (75 RDA) toothpaste was used, with a soft toothbrush and a standardized brushing force of 1.5 N by using a mechanical brushing device. The direct light‐cured materials containing nanofillers (Filtek Supreme) or fumed silica and silica glass dispersed into the matrix (G‐aenial Universal) resisted better to this test than the hybrid composites and even than their relative CAD/CAM blocks. This rather surprising behavior could be partially due to a possible bias of this study as the time of renewing the toothpaste slurry was set to 5 min. Containing large amounts of rounded nano‐particles, the detachment of such fillers could have left a more favorable topography than the hybrid materials (Tetric EvoCeram and GrandioSO x‐tra), and detached nano‐fillers could have also acted as an additional polishing agent. In the particular cases of GrandiSO x‐tra and Grandio CAD that showed the lowest gloss values in this test, a possible explanation could be related to a sub‐optimal silanization of the filler particles which could lead to the so‐called pothole effect after detachment, creating large surface cavitations resulting in huge gloss decrease. Our results are substantially in accordance with Ardu et al. (2020), and slightly different from Lefever et al. (2012, 2014) who did similar research. These differences can be easily explained by the fact that we performed manual polishing of the samples with Sof‐Lex discs while they did it by means of a polishing machine which allowed for higher initial gloss values.

Distilled water was used a negative control in the present study. In particular, samples of all tested materials were dipped in distilled water for 1 h and measured before and after the immersion. Gloss values were almost identical and, obviously, no statistical differences were detected between values before and after aging in distilled water.

When judging on the clinical relevance of gloss variation in the three experiments, not only statistical differences have to be taken into account but also their clinical implications. According to Tessarin et al. (2018) ΔGU of 17.6 units is the limit of perceptibility of gloss variation, which is defined as the probability of at least 50% of observers to detect a gloss difference. Based on this criterium, alcohol did not affect human gloss perception with all materials tested, while Elmex gelée perceptibly affected all tested materials with the only exception of Cerasmart 270, and the brushing test caused clinically perceptible changes in all the tested materials.

Further in vitro and in vivo research with other direct composite as well as CAD/CAM blocks are needed in order to confirm these findings.

The null hypothesis that mechanical and chemical agents do not decrease surface gloss of direct composite resin materials as well as indirect CAD/CAM blocks was rejected.

## 5 CONCLUSIONS

Gloss retention seems to be dependent on the composite type (direct or CAD/CAM block) and composite brand and varies in respect to the type of aging. CAD/CAM materials showed a higher resistance toward alcohol exposure. However, these results must be interpreted with caution as they are linked to the specificity of the experiment's setting.

## CONFLICT OF INTEREST

The authors declare that there is no conflict of interest.

## AUTHOR CONTRIBUTIONS

All authors contributed to the study conception and design. Material preparation, data collection, and analysis were performed by Olivier Duc, Emilie Bétrisey, Enrico Di Bella, René Daher. The first draft of the article was written by Stefano Ardu, corrected and integrated by Ivo Krejci and all authors commented on the subsequent versions of the article. All authors read and approved the final article.

## ETHICAL APPROVAL

This article does not contain any studies with human participants or animals performed by any of the authors.

## Data Availability

We would like that data openly available in a public repository that issues datasets with DOIs.
